# Caravan - A global community dataset for large-sample hydrology

**DOI:** 10.1038/s41597-023-01975-w

**Published:** 2023-01-31

**Authors:** Frederik Kratzert, Grey Nearing, Nans Addor, Tyler Erickson, Martin Gauch, Oren Gilon, Lukas Gudmundsson, Avinatan Hassidim, Daniel Klotz, Sella Nevo, Guy Shalev, Yossi Matias

**Affiliations:** 1 Google Research, Vienna, Austria; 2 Google Research, Mountain View, CA USA; 3 Fathom, Square Works, Bristol, UK; 4 Geography, University of Exeter, Exeter, UK; 5 Google, Mountain View, CA USA; 6 Institute for Machine Learning, Johannes Kepler University, Linz, Austria; 7 Google Research, Tel Aviv, Israel; 8 Institute for Atmospheric and Climate Science, ETH Zurich, Zurich, Switzerland

**Keywords:** Hydrology, Water resources

## Abstract

High-quality datasets are essential to support hydrological science and modeling. Several CAMELS (Catchment Attributes and Meteorology for Large-sample Studies) datasets exist for specific countries or regions, however these datasets lack standardization, which makes global studies difficult. This paper introduces a dataset called *Caravan* (a series of CAMELS) that standardizes and aggregates seven existing large-sample hydrology datasets. Caravan includes meteorological forcing data, streamflow data, and static catchment attributes (e.g., geophysical, sociological, climatological) for 6830 catchments. Most importantly, Caravan is both a dataset and open-source software that allows members of the hydrology community to extend the dataset to new locations by extracting forcing data and catchment attributes in the cloud. Our vision is for Caravan to democratize the creation and use of globally-standardized large-sample hydrology datasets. Caravan is a truly global open-source community resource.

## Background & Summary

Data underpin our understanding of the storage and transport of water at the Earth’s surface. Hydrological processes (e.g., streamflow generation) are governed by hydroclimatic variables (e.g., rainfall, temperature, humidity) and landscape characteristics (e.g., soils, landcover, human intervention). These interactions govern the availability of water resources and the occurrence of extreme events like floods and droughts.

Detailed datasets combining hydroclimatic time series, landscape attributes, and/or hydrological response variables like streamflow exist for many experimental catchments, in many cases spanning decades^1–3^. However, it is not possible to capture the diversity of hydrological behavior from any individual watershed. In parallel, there also exist tens of thousands of gauges monitoring rivers across the world. Although data available from these gauges are limited in that they do not describe all of the hydrological processes in a given watershed, the large number of gauges means that they cover a wide of range of hydrological regimes and extreme events^4–7^. Gupta *et al*.^8^ argued that large sample sizes allow for assessment of the generality of hydrological models and research findings. Large sample sizes also allow for large-scale research like detecting and attributing systematic shifts in terrestrial water availability at regional^9,10^ to global scales^11,12^. Moreover, large sample datasets are necessary for developing generalizable data-driven models^13–16^.

Recognizing this has led to the development of a sub-discipline in the hydrological sciences called *large-sample hydrology* (LSH), which relies on data from hundreds to thousands of catchments^17^. There are an increasing number of publicly available LSH datasets. Arguably, the first open LSH dataset was from the Model Parameter Estimation Experiment (MOPEX)^18^, which contains data from 431 basins within the United States through 2003. Later datasets were developed for specific countries or regions, including Australia^19^, Austria^20^, Brazil^21^, North-America^22^, China^23^, Chile^24^, Europe^25^, Great Britain^26^, Thailand http://hydro.iis.u-tokyo.ac.jp/GAME-T/GAIN-T/routine/rid-river/index.html, the United States^27,28^, and the Arctic https://www.r-arcticnet.sr.unh.edu/v4.0/index.html. Many of these are referred to as *Catchment Attributes and MEteorology for Large-sample Studies (CAMELS)* datasets^19,21,24,26,28^.

Although none of the existing CAMELS datasets are global, there are global collections of streamflow data like the Global Streamflow Indices and Metadata Archive (GSIM)^29,30^, which provides monthly and seasonal streamflow indices for 35,000+ locations, and the Global Runoff Data Base https://www.bafg.de/GRDC, which provides river discharge estimates at 10,000+ locations. Both of these collections, however, are not coupled with catchment attributes or meteorological forcing data. Critically, GSIM does not provide daily streamflow data (only indices), and GRDC does not allow for redistribution of raw data, which makes it difficult to standardize with other datasets. Furthermore, although data from 10,000+ stations are available through GRDC, both the quality of the available records and the period of record for individual basins varies significantly^30^. On the other hand, HydroATLAS^31^ provides global catchment attributes, but does not include meteorological or streamflow data. There are also proprietary or non-public hydrological datasets that have been used for hydrological research–for example, datasets used by Beck *et al*.^32,33^, for global model calibration or by Blöschl *et al*.^34^ for extrapolating climate change impacts on flooding (less than a third of one percent of the daily time series used in the latter study are publicly available, last access 20th March 2022). There are many reasons why proprietary datasets exist in today’s research landscape. These often encompass causes that lie outside the domain of influences of individual research groups. However, from a scientific perspective, proprietary datasets are a roadblock to open, collaborative, reproducible, and extensible research.

Aside from the fact that no comprehensive, global LSH dataset exists, Addor *et al*.^17^ identified four major limitations of many of the existing region-specific datasets: (i) lack of common standards to allow for intercomparison, (ii) lack of metadata and uncertainty estimates to assess data reliability, (iii) lack of information about human interventions, and (iv) limited accessibility. Addor *et al*.^17^ also outlined desiderata for standardizing and automating the development of LSH datasets, including (i) basic data requirements, (ii) naming conventions for hydrologically-relevant variables, (iii) publicly available data processing code, (iv) uncertainty estimates, (v) anthropogenic descriptors, and (vi) adhering to FAIR data standards^35^. They propose that community, cloud-based infrastructure could help overcome these limitations, by allowing for the use and development of standardized practices and codebases.

The *Caravan* dataset presented here is a step toward realizing this vision. The basis for Caravan is a collection of region-specific datasets, which are merged and standardized in a way that is designed with the following characteristics:

1. Standardized: Data are standardized globally meaning that the same meteorological and landscape variables exist for all catchments, and are derived using the same procedures from the same source datasets.
2. Open: All data are publicly available with an open license.
3. Extensible: All software tools and source datasets used to produce Caravan are open and accessible through a cloud platform (Google Earth Engine) to enable others to extend (i.e., add catchments to) the dataset.

**Fig. 1:**
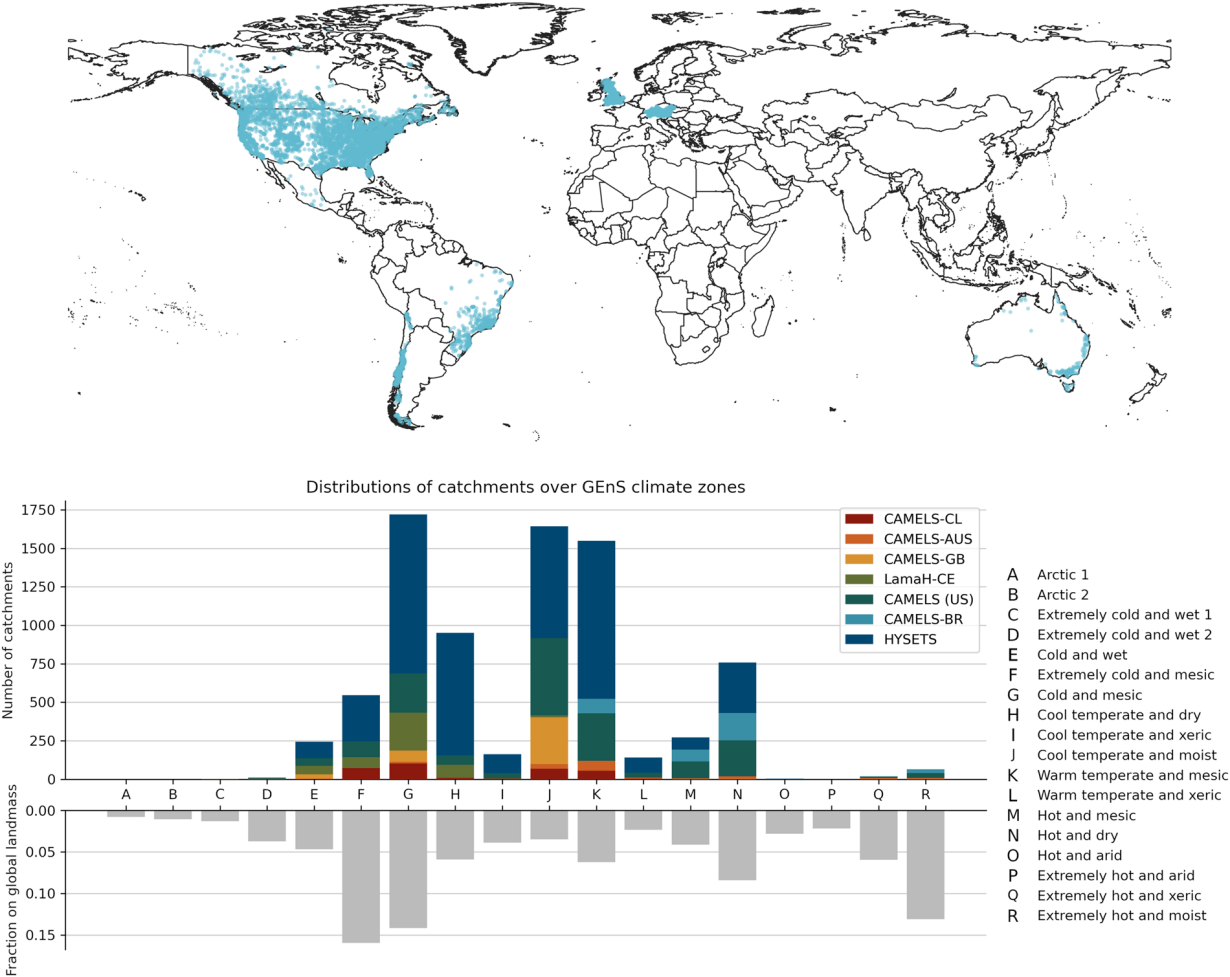
Top: Global distribution of catchments included in Caravan. Bottom: Distribution of the 6830 Caravan catchments among the Global Environmental Stratification (GEnS) climate zones. The bottom part of the plots shows the fraction of a particular climate zone on the total land mass.

**Fig. 2:**
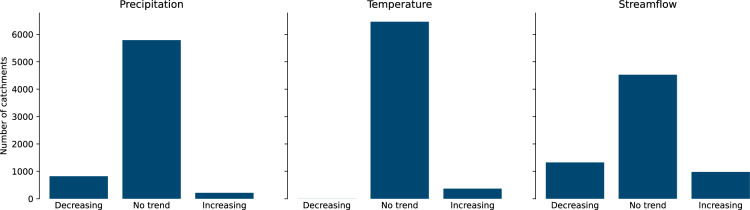
Number of catchments in Caravan (6830 basins over ~40 years of data) with statistically significant (*α* = 0.05) trends in three variables: mean temperature, precipitation, and discharge, assessed by an unmodified Mann-Kendall test. All data were averaged monthly before computing statistical trends.

The third point is especially important. Most streamflow gauges are maintained by local or national organizations, and the data from these gauges are rarely FAIR (Findable, Accessible, Interoperable and Re-usable). Caravan is designed to be extensible, so that anyone can easily derive meteorological forcings and landscape attributes for additional catchments using a standardized procedure. This allows new catchments to be used in the context of this larger dataset (e.g., for training models, assessing relative climate impacts, etc.), and it allows organizations with streamflow data from any number of catchments (from one to thousands) to quickly and easily add their data to the larger public Caravan dataset in a way that is standardized with all other catchment data. Our vision is for Caravan to be the platform for a larger community data resource–we see this as perhaps the most direct path to developing a truly open global hydrological dataset. The current Caravan dataset that we introduce here includes streamflow observations from 6830 basins, spanning most Global Environmental Stratification (GEnS) climate zones^36^, with the exception of arctic, extreme cold, and arid zones (Fig. 1). Caravan includes daily data from almost four decades (1981–2020), including catchments that experienced significant climate trends (Fig. 2).

## Methods

### Basin selection & streamflow data

Daily streamflow observations for the 6830 basins currently in Caravan were aggregated from several existing open datasets:

- 482 basins from CAMELS (US)^27^
- 150 basins from CAMELS-AUS^19^
- 376 basins from CAMELS-BR^21^
- 314 basins from CAMELS-CL (using an updated Version from January 2022)^24^
- 408 basins from CAMELS-GB^26^
- 4621 basins from HYSETS^22^
- 479 basins from LamaH-CE^20^

These datasets were selected because (i) they include catchment boundaries for each streamflow gauge, and (ii) because their licenses allow redistribution. Furthermore, we currently only include basins equal or larger than 100 km^2^ and smaller than 2000 *km*^2^. Streamflow data is normalized by catchment area to units of *mm/day*. All data are reported in the local time zone (non-daylight saving time for the entire year) of the gauge station, which is included in metadata.

**Fig. 3:**
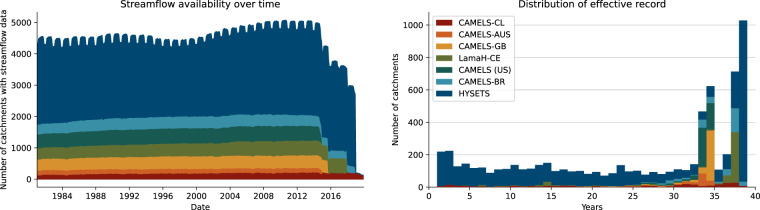
Density of active Caravan gauge records through time (left) and distribution of water-years worth of data from each of 6830 basins in Caravan (right).

Time periods of available streamflow observations varies between basins, however we did not include any streamflow data prior to 1981 because this is the beginning of the ERA5-Land reanalysis, which was used to derive meteorological forcing data. Figure 3 shows density of streamflow records through time (left) and the distribution of lengths of daily streamflow records (right), emphasizing that comparatively long flow time series are available for the Caravan catchments (the median length is 31 years).

### Meteorological forcing data

Caravan includes meteorological forcing data from ERA5-Land^37^. This choice was made for the following reasons:

- Global coverage and spatial consistency: Although ERA5-Land data products are often lower-accuracy (i.e., more uncertain) than local, high-resolution meteorological data sets, only globally available data sets allow for comparative studies at a global scale.
- Sub-daily (e.g., hourly) resolution: All daily average streamflow observations in the source datasets are reported in the corresponding local time of the gauge station. In contrast, global meteorological data products are usually provided in GMT + 0. To be able to calculate the matching daily average meteorological forcing data for the daily averaged streamflow observation, it is therefore necessary to have sub-daily meteorological data, so that we can shift the meteorological data according to the local time zone of the gauge station, before computing daily aggregates.
- Availability in the cloud: one of our goals was to do all heavy computing tasks in the cloud (here: Google Earth Engine). ERA5-Land provides hourly data on Google Earth Engine.
- Permissive license: A core principle of Caravan is to democratize LSH datasets and dataset development. ERA5-Land has a permissive license that allows free distribution.

ERA5-Land meteorological variables used in Caravan are listed in Table 1–these are typical variables used as forcing data (or boundary conditions) for hydrology and land surface models. We first computed the area-weighted spatial average for each variable in each catchment area from hourly spatial data (~9*km* spatial resolution) and shifted the hourly time series (natively at GMT + 0) to the local time of each gauge. We then computed different daily statistics for each variable according to the Aggregation column in Table 1.

**Table 1:** ERA5-Land meteorological variables. Daily aggregates are computed in local time of each basin.

Feature (ERA5-Land variable name)	Aggregation	Unit
Precipitation (total_precipitation)	Daily sum	*mm/day*
Potential evaporation (potential_evaporation)i	Daily sum	*mm/day*
Air temperature (temperature_2m)	Daily min/max and mean	°*C*
Dew point temperature (dewpoint_temperature_2m)	Daily min/max and mean	°*C*
Shortwave radiation (surface_net_solar_radiation)	Daily min/max and mean	*Wm*^−2^
Net thermal radiation at the surface (surface_net_thermal_radiation)	Daily min/max and mean	*Wm*^−2^
Surface pressure (surface_pressure)	Daily min/max and mean	*kPa*
Eastward wind component (u_component_of_wind_10m)	Daily min/max and mean	*ms*^−1^
Northward wind component (v_component_of_wind_10m)	Daily min/max and mean	*ms*^−1^

i: Be cautious with these values as they include unrealistically high values, see also^20^.

### Reference model states

In addition to meteorological forcing data, Caravan includes time series of modeled soil moisture and snow states from ERA5-Land (Table 2). These time series are included to provide reference values or benchmark values for studies that analyze or model hydrological states. These time series data were processed in the same way as meteorological forcing data.

**Table 2:** ERA5-Land model state variables. Daily aggregates are computed in local time of each basin.

Feature (ERA5-Land variable name)	Aggregation	Unit
Snow water equivalent (snow_depth_water_equivalent)	Daily min/max and mean	*mm*
Soil water volume 0–7 cm (volumetric_soil_water_layer_1)	Daily min/max and mean	*m*^3^*/m*^3^
Soil water volume 7–28 cm (volumetric_soil_water_layer_2)	Daily min/max and mean	*m*^3^*/m*^3^
Soil water volume 28–100 cm (volumetric_soil_water_layer_3)	Daily min/max and mean	*m*^3^*/m*^3^
Soil water volume 100–289 cm (volumetric_soil_water_layer_4)	Daily min/max and mean	*m*^3^*/m*^3^

### Catchment attributes

Caravan includes two sets of catchment attributes: (i) attributes derived from HydroATLAS^31,38^ and (ii) climate attributes derived from the daily ERA5-Land time series included in Caravan. The latter are similar to the climate attributes provided in CAMELS-US^28^. The reasons for choosing HydroATLAS as the source for the former are similar to the reasons for choosing ERA5-Land for time series data: HydroATLAS has global coverage with a license that allows for redistribution.

The catchment attributes derived from HydroATLAS use the highest resolution shape file available in that dataset (level 12). The level 12 HydroATLAS polygons are, for the vast majority of basins, smaller than the catchment boundaries for each gauge station provided by the respective CAMELS datasets–i.e., a single polygon representing the drainage area for a specific gauge include multiple HydroATLAS polygons. Therefore, we first computed the spatial join of the HydroATLAS polygons and the catchment boundaries and then derived the catchment attributes as an area-weighted aggregate (see the Aggregation column in Tables 3, 4). Catchment attributes included in Caravan can be loosely grouped into the following categories: hydrology, physiography, climatology, soils & geology, land cover characteristics, and anthropogenic influences. A full list of all catchment attributes derived from HydroATLAS is given in Tables 3–5 contains a list of attributes that were derived from ERA5-Land time series. Lastly, Table 6 lists additional attributes that are also included in Caravan, such as the latitude and longitude coordinates of each gauge station, the station name, the country of the gauge station location and the catchment area.

**Table 3:** First set of catchment attributes derived from HydroATLAS that are included in Caravan from the groups Hydrology, Physiography, and Climate.

Group	Description (HydroATLAS name)	Aggregation	Unit
Hydrology	Natural discharge (dis_m3_p[mn, mx,yr])	annual min/max/mean	*m*^3^ s^−1^
	Land surface runoff (run_mm_syr)	spatial mean of sub-basin runoff	*mm*
	Inundation extent (inu_pc_s[mn, mx, lt])	annual min/mean and long-term max	%
	Limnicity - percent lake area (lka_pc_sse)	spatial extent	%
	Lake Volume (lkv_mc_usu)	at reach pour point	10^6^ *m*^3^
	Reservoir volume (rev_mc_usu)	at reach pour point	10^6^ *m*^3^
	Degree of regulation (dor_pc_pva)	index at reach pour point	
	River area (ria_ha_ssu)	at reach pour point	*hectares*
	River volume (ria_tc_ssu)	at reach pour point	10^3^ *m*^3^
	Groundwater table depth (gwt_cm_sav)	spatial mean	*cm*
Physiography	Elevation (ele_mt_s[av, mn, mx])	spatial mean/min/max	*m* above sea level
	Terrain slope (slp_dg_sav)	spatial mean	° (x10)
	Stream gradient (sgr_dk_sav)	mean of reach segments	*dm*/*km*
Climate	Climate zones from GEnS (clz_cl_smj)	spatial majority	classes (n = 18)
	Climate strata from GeNS (cls_cl_smj)	spatial majority	classes (n = 125)
	Air temperature (tmp_dc_s[01–12, mn, mx, yr])	monthly mean, annual mean/min/max	°*C* (x10)
	Precipitation (pre_mm_s[01–2, yr])	monthly mean, annual mean	*mm*
	Potential evapotranspiration (pet_mm_s[01–12, yr])	monthly mean, annual mean	*mm*
	Actual evapotranspiration (aet_mm_s[01–12, yr])	monthly mean, annual mean	*mm*
	Global aridity index (ari_ix_sav)	spatial mean	index value (x10)
	Climate moisture index (cmi_ix_s[01–12, yr])	monthly mean, annual mean	index value (x10)
	Snow cover extent (snw_pc_s[01–12, mx, yr])	monthly mean, annual max/mean	% cover

**Table 4:** Second set of catchment attributes derived from HydroATLAS that are included in Caravan from the groups Land Cover, Soils & Geology, and Anthropogenic.

Group	Description (HydroATLAS name)	Aggregation	Unit
Land Cover	Land cover classes (glc_cl_smj)	spatial majority	classes (n = 22)
	Land cover extent (glc_pc_s[01–22])	spatial mean	% cover
	Potential natural vegetation classes (pnv_cl_smj)	spatial majority	classes (n = 15)
	Potential natural vegetation extent (pnv_pc_s[01–15])	spatial mean	% cover
	Wetland classes (wet_cl_smj)	spatial majority	classes (n = 12)
	Wetland extent (wet_pc_s[01–09, g1, g2])	spatial mean	% cover & grouping
	Forest cover extent (for_pc_sse)	spatial mean	% cover
	Cropland extent (crp_pc_sse)	spatial mean	% cover
	Pasture extent (pst_pc_sse)	spatial mean	% cover
	Irrigated area extent (equipped) (ire_pc_sse)	spatial mean	% cover
	Permafrost extent (prm_pc_sse)	spatial mean	% cover
	Protected area extent (pac_pc_sse)	spatial mean	% cover
	Terrestrial biomes (tbi_cl_smj)	spatial majority	classes (n = 14)
	Terrestrial ecoregions (tec_cl_smj)	spatial majority	classes (n = 846)
	Freshwater major habitat types (fmh_cl_smj)	spatial majority	classes (n = 13)
	Freshwater ecoregions (fec_cl_smj)	spatial majority	classes (n = 426)
Soils & Geology	Clay fraction in soil (cly_pc_sav)	spatial mean	%
	Silt fraction in soil (slt_pc_sav)	spatial mean	%
	Sand fraction in soil (snd_pc_sav)	spatial mean	%
	Organic carbon content in soil (soc_th_sav)	spatial mean	tonnes/hectare
	Soil water content (swc_pc_s[01–12, yr])	monthly mean, annual mean	%
	Lithological classes (lit_cl_smj)	spatial majority	classes (n = 16)
	Karst area extent (kar_pc_sse)	spatial mean	% cover
	Soil erosion (ero_kh_sav)	spatial mean	*kg*/*hectare*/*yr*
Anthropogenic	Population count (pop_ct_usu)	at reach pour point	count (thousands)
	Population density (ppd_pk_sav)	spatial mean	people per *km*^2^
	Urban extent (urb_pc_sse)	spatial mean	% cover
	Nighttime lights (nli_ix_sav)	spatial mean	index value (x100)
	Road density (rdd_mk_sav)	spatial mean	*m/km*^2^
	Human footprint (hft_ix_s[93,09])	spatial mean for 1993 & 2009	index value (x100)
	Gross domestic product (gdp_ud_sav)	spatial mean	USD ($)
	Human development index (hdi_ix_sav)	spatial mean	index value (x1000)

**Table 5:** Climate attributes derived from ERA5-Land time series.

Attribute	Description	Unit	Reference
p_mean	Mean daily precipitation	*mm/day*	
pet_mean	Mean daily potential evaporation	*mm/day*	
aridity	Aridity index, ratio of mean PET and mean precipitation	—	
frac_snow	Fraction of precipitation falling as snow	—	^59^
moisture_index	Mean annual moisture index in range [−1, 1], where −1 indicates water-limited conditions and 1 energy-limited conditions	—	^59^
seasonality	Moisture index seasonality in range [0, 2], where 0 indicates no changes in the water/energy budget throughout the year and 2 indicates a change from fully arid to fully humid.	—	^59^
high_prec_freq	Frequency of high precipitation days, where precipitation ≥5 times mean daily precipitation	—	^28^
high_prec_dur	Average duration of high precipitation events (number of consecutive days where precipitation ≥5 times mean daily precipitation	days	^28^
low_prec_freq	Frequency of low precipitation days, where precipitation <1 mmday-1	—	^28^
low_prec_dur	Average duration of low precipitation events (number of consecutive days where precipitation <1 mmday-1	days	^28^

**Table 6:** Metadata and other attributes.

Attribute	Description	Unit
gauge_lat	Latitude coordinate of the gauge	—
gauge_lon	Longitude coordinate of the gauge	—
gauge_name	Station name	—
country	Country of the gauge location	—
area	Catchment area	*km*^2^

### Data processing in the cloud

The major computational challenge for developing LSH datasets is processing gridded meteorological and attributes data. To make the development and augmentation of Caravan as democratic as possible (i.e., to make it as easy as possible for anyone to add new watersheds or new data layers to the dataset), all of our data processing scripts use Google Earth Engine via Python APIs. Google Earth Engine^39^ is a free-to-use cloud service with a large catalogue of geospatial data, including all of the datasets described above. The Caravan data processing scripts interact with Earth Engine directly through APIs, so that there is no need for individuals to download data from Earth Engine outside of these scripts. This has two benefits: it is not necessary for users to download and store large amounts of gridded meteorological data, and does not require any specific hardware. Any individual hydrologist, modeler, researcher, or student should be able to process even large numbers of new watersheds with minimal effort or expense. All that is necessary to add a new gauge to the Caravan dataset is a shapefile representing the drainage area of the catchment, plus a timeseries of daily or subdaily streamflow (discharge) values from that gauge in local time. Instructions about how to add new catchments to Caravan are provided in a Readme file in the dataset repository.

## Data Records

The current version of the Caravan dataset (6830 watersheds)^40^ is available at 10.5281/zenodo.7540792. A project homepage is available at https://github.com/kratzert/Caravan/, including all code and where news and updates are announced.

The dataset is organized into the following subfolders:

- The *attributes* folder contains one subfolder per source dataset, which each contain two csv (comma separated values) files. One file (‘attributes_hydroatlas_{source}.csv’) contains attributes derived from HydroATLAS and the other file (‘attributes_caravan_{source}.csv’) contains limate indices derived from ERA5-Land, where {source} indicates the corresponding source data set (e.g. *camelsgb* for CAMELS-GB, *camelscl* for CAMELS-CL, and so on). The first column in all attributes file is called ‘gauge_id’ and contains a unique basin identifier of the form ‘{source}_{id}’, where {source} again is the abbreviation of the corresponding source dataset, and {id} is the basin id as defined in the original source dataset.
- The *shapefiles* folder contains one subfolder per source dataset. Each of these subfolders contains a shapefile with the catchment boundaries of each basin within that dataset. These are the shapefiles that were used to derive the catchment attributes and ERA5-Land time series data. Each polygon in a given shapefile has a field ‘gauge_id’ that contains the unique basin identifier.
- The *timeseries* folder contains two subfolders, *csv* and *netcdf*, that both share the same structure and contain the same data, once as csv-files and once as netCDF files. Each of these two subfolders contains one subfolder per source dataset. Within these source dataset specific subdirectories, there is one file (either csv or netCDF) per basin, containing all time series data (meteorological forcings, state variables, and streamflow). The netCDF files also contain metadata information, including physical units, timezones, and information on the data sources.
- The *code* folder contains all scripts and Jupyter notebooks that were used to derive the data set. These scripts can be used to extend the data set to any new basin in the world. Instructions are included in the README.md file contained in this folder.
- The *licenses* folder contains license information of all data included in Caravan and for Caravan itself. General license information are listed in the README.md file in this directory, source dataset specific information are listed in the files located in the source dataset specific subdirectories.
- The *README.md* file in the main directory includes a description of the dataset structure, information on the units of time series data, and time zones.

All time series data except streamflow are aggregated (daily and spatially over basins) from ERA5-Land. ERA5-Land is available directly from^41^, however we used the Google Earth Engine repository. HydroATLAS attributes were derived from the HydroATLAS dataset^42^. Streamflow time series are collected from the respective region-specific repositories: Australia^43^, Brazil^44^, Canada^22^, Chile^45^, Great Britain^46^, LamaH-CE (Austrian territory and Danube catchment up to Bratislava)^47^, and the United States:^48^.

## Technical Validation

### Aggregating HydroATLAS attributes

**Fig. 4:**
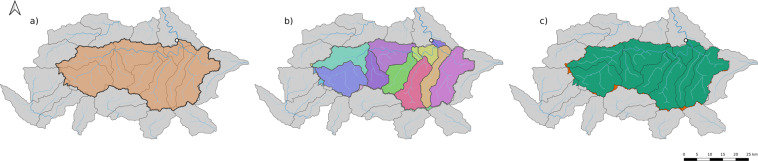
Visualization of the process of selecting HydroATLAS polygons for deriving catchment attributes for one randomly selected catchment. (**a**) The orange polygon (bold outline) is the catchment of interest, as represented by a shapefile from one of the CAMELS datasets. Grey polygons (thin outlines) are HydroATLAS (level 12) polygons of the surrounding area. The white dot denotes the catchment outlet (gauge location) and blue lines denote the river network. (**b**) Shows all HydroATLAS polygons or subsections of HydroATLAS polygons that intersect with the catchment polygon. Note that due to different underlying digital elevation maps, the boundaries of the polygons do not match perfectly. This leads to small intersection artifacts at catchment boundary. To alleviate this problem we excluded small polygons (smaller than 5km2) when deriving the area weighted catchment attributes from HydroATLAS. c) Shows the excluded (orange) intersecting polygons and the area used for deriving attributes (green).

The majority of catchment attributes are derived from HydroATLAS. The key challenge in extracting data from HydroAtlas is to define which HydroATLAS polygons are within a given gauge’s drainage area. The primary complication is that all datasets–i.e., the various CAMELS datasets and HydroATLAS use shapefiles derived from different digital elevation maps (DEM) at different spatial resolution. This means that catchment boundaries from the source datasets do not perfectly align with the polygons in HydroATLAS. An example of this is shown in Fig. 4. This figure shows the drainage area for a particular gauge, as specified by the shapefile in the CAMELS dataset (first subpanel), the collocated HydroATLAS subbasin polygons (second panel), and the mismatch between the two due to different datasets deriving catchment boundaries from different DEMs (third panel).

**Fig. 5:**
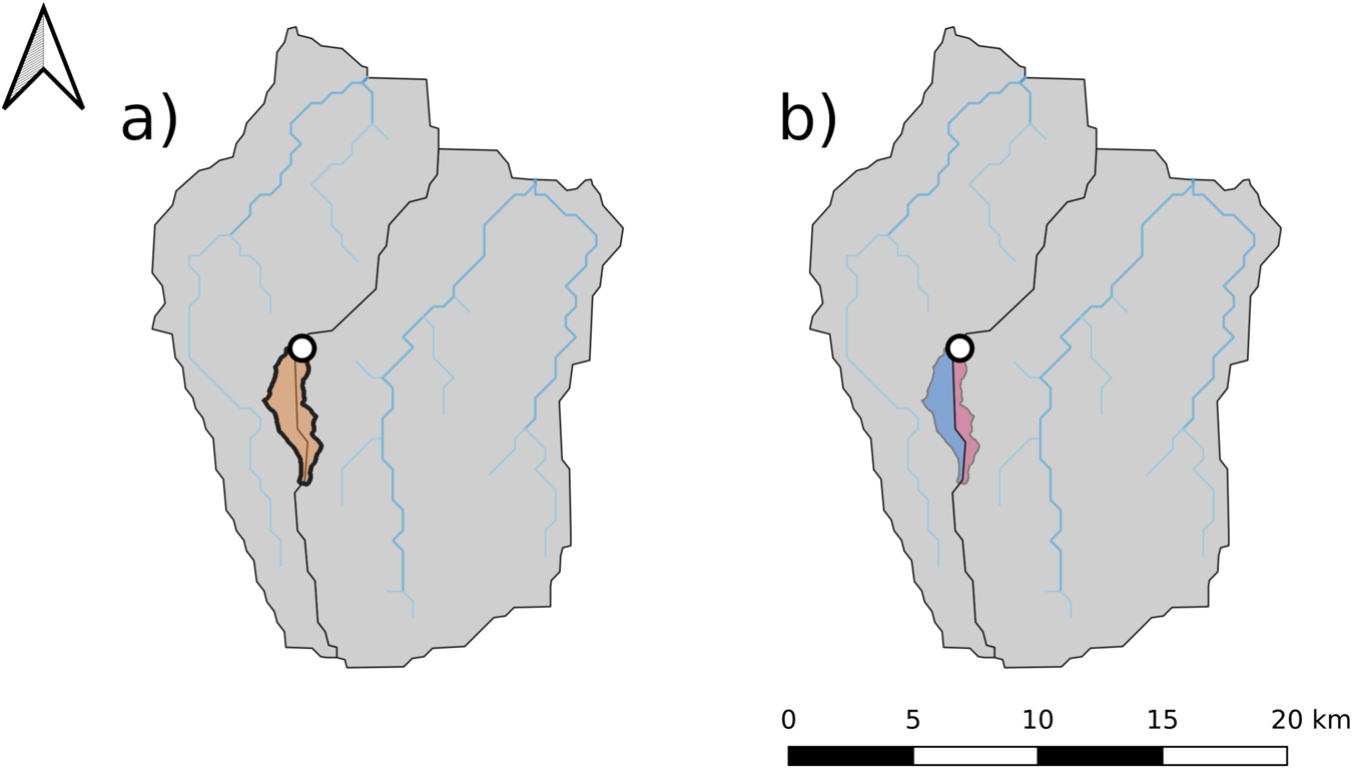
Example of small basin that was excluded from the dataset. (**a**) The orange polygon (bold outline) denotes the catchment, the two grey polygons (thin outlines) are the surrounding HydroATLAS polygons, and the white dot denotes the catchment outlet. (**b**) Shows the two intersecting areas of the HydroATLAS polygons with the catchment area. Both areas are a) smaller the minimum intersection area explained in Fig. 4b) from looking at the gauge location, it can be seen that the larger of the two intersections (blue) is in the neighboring HydroATLAS polygon that should not contribute when deriving the catchment attributes.

**Fig. 6:**
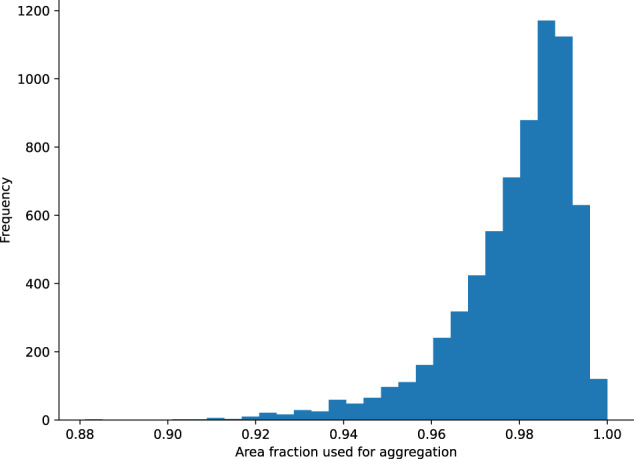
Histogram showing the fraction of the catchment area that is considered when aggregating the HydroATLAS attributes across all basins. Considering Fig. 4c, this value is computed as the fraction of the green area by the sum of the green and orange area.

Because of this mismatch along catchment boundaries between different watershed delineations in different datasets, we chose to only include gauges with total drainage areas of at least 100 *km*^2^. In smaller catchments, this boundary effect can represent a significant fraction of the total area of the catchment–an example of this is illustrated in Fig. 5. To quantify this area mismatch, we included a static feature called *area_fraction_used_for_aggregation*, which is the fraction of the area used for the aggregation and the total catchment area. In Fig. 4c, this would be the fraction of the green area by the sum of the green and orange areas. The distribution of these values across all basins is shown in Fig. 6.

### Validating meteorological time series

Like most data about the natural environment, hydrological data is typically associated with significant uncertainty. Quantifying uncertainty is a central part of hydrological research^49,50^, and usually involves intensive field campaigns^51,52^, statistical comparison between several data products^53–55^, or modeling studies^56,57^–all of which are outside the scope of the current project. We can, however, statistically verify the processing tools that were used to develop the Caravan data from existing datasets. We did this verification by comparing Caravan-derived meteorological forcings (from ERA5–Land) with forcings from CAMELS-US. CAMELS-US was chosen because it includes three independent meteorological data sources (NLDAS, Maurer, DayMet), which allows us to contextualize the variability between CAMELS-US forcings and Caravan forcings. There will always be some amount of variability between any two meteorological datasets, and having three meteorological data products allows us to contextualize any variability between Caravan features and CAMELS-US features.

**Fig. 7:**
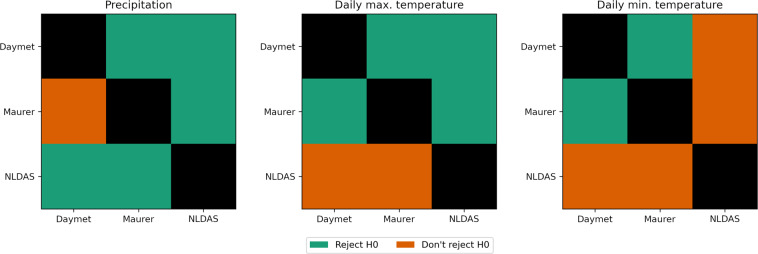
Results of one-way, paired t-tests with the null hypothesis (H0) that per-basin correlation coefficients between Caravan meteorological data and any given CAMELS-US meteorological data product (NLDAS, DayMet, Maurer) are not significantly lower than per-basin correlation coefficients between a given pair of CAMELS-US meteorological data products. The null hypothesis for the test in each grid cell compares correlations between Caravan and the CAMELS-US data product on the y-axis vs. correlations between the CAMELS-US data products on the x- and y-axes. Rejecting the null hypothesis indicates that the Caravan-related correlations are significantly lower than the correlations between the two CAMELS-US products (*α* = 0.9).

We calculated the correlation (Pearson r) between each pair of forcing data products (NLDAS, Maurer, DayMet, ERA5-Land) separately in each basin (n = 482) for three meteorological variables: total daily precipitation and daily maximum and minimum temperatures. We then used a set of one-tailed, paired t-tests to test hypotheses that for each of the three meteorological variables, correlations between Caravan and any individual CAMELS-US data product were significantly (*α* = 0.90) lower than correlations between each pair of CAMELS-US forcing products. Figure 7 shows the results of these tests. Although certain forcings are more highly correlated than others (e.g., DayMet and Maurer are more highly correlated than DayMet and NLDAS), correlations between Caravan and CAMELS-US data products were not consistently lower than correlations between different CAMELS-US data products.

## Usage Notes

Our vision for Caravan is as the foundation of a dynamically growing community LSH dataset that anyone in the hydrology community can access and augment. Currently, the spatial distribution of basins included in Caravan is limited to a few regions in the world, see Fig. 1. We hope that some users will be willing (and allowed) to share their data, so that Caravan, over time, will contain discharge data from most parts of the world. In fact, while this manuscript was in review, a community extension was provided, adding 308 basins from Denmark^58^. Detailed instructions for adding new catchments to Caravan are provided in the dataset repository, as well as in the code repository. This includes all code necessary to derive meteorological and attributes data on Google Earth Engine for any new basin globally. All computation can be done for free using Google Earth Engine.

In the introduction, we noted that Addor *et al*.^17^ listed six desiderata for LHS datasets. Caravan meets five of those six criteria–the missing desideratum is to have uncertainty estimates on all data components. Assessing uncertainty in hydrological data is difficult without relying on strong assumptions (often, some type of hydrological model), and we expect that future work will apply various methods for quantifying the uncertainty in global rainfall-runoff datasets. Perhaps that a comparison of the attributes and timeseries provided in Carvan, and those from the LSH original datasets, could provide new insights into their uncertainty, and inform the selection of datasets for hydrology.

## Data Availability

The code that was used to produce the Caravan dataset is available at https://github.com/kratzert/Caravan/.
